# Quantitative assessment of intestinal stiffness and associations with fibrosis in human inflammatory bowel disease

**DOI:** 10.1371/journal.pone.0200377

**Published:** 2018-07-11

**Authors:** Daniel C. Stewart, Dalton Berrie, Jian Li, Xinyue Liu, Cooper Rickerson, David Mkoji, Atif Iqbal, Sanda Tan, Andria L. Doty, Sarah C. Glover, Chelsey S. Simmons

**Affiliations:** 1 J. Crayton Pruitt Family Department of Biomedical Engineering, Herbert Wertheim College of Engineering, University of Florida, Gainesville, FL, United States of America; 2 Division of Gastroenterology, Hepatology and Nutrition, College of Medicine, University of Florida, Gainesville, FL, United States of America; 3 Department of Pharmaceutical Outcomes and Policy, College of Pharmacy, University of Florida, Gainesville, FL, United States of America; 4 Department of Mechanical and Aerospace Engineering, Herbert Wertheim College of Engineering, University of Florida, Gainesville, FL, United States of America; 5 Department of Surgery, College of Medicine, University of Florida, Gainesville, FL, United States of America; 6 Division of Cardiovascular Medicine, College of Medicine, University of Florida, Gainesville, FL, United States of America; National Cancer Institute, UNITED STATES

## Abstract

Inflammatory bowel disease (IBD) continues to increase in prevalence in industrialized countries. Major complications of IBD include formation of fibrotic strictures, fistulas, reduced absorptive function, cancer risk, and the need for surgery. In other chronic gastrointestinal disease models, stiffness has been shown to precede fibrosis; therefore, stiffness may be a reasonable indicator of progression toward stricture formation in IBD patients. Herein, we seek to quantify tissue stiffness and characterize fibrosis in patients with IBD and to compare mechanical properties of unaffected human tissue to common animal species used for IBD studies. Inflamed and unaffected tissue from IBD patients and unaffected tissue from mice, pigs, and cows were indented using a custom device to determine the effective stiffness. Histology was performed on matched tissues, and total RNA was isolated from IBD tissue samples and used for gene expression analysis of pro-fibrotic genes. We observed an increase in the effective stiffness (steady-state modulus, SSM) (p < 0.0001) and increased expression of the collagen type I gene (COL1A1, p = 0.01) in inflamed tissue compared to unaffected areas in our IBD patient cohort. We also found that increased staining of collagen fibers in submucosa positively correlated with SSM (p = 0.093). We determined that unaffected animal bowel stiffness is significantly greater than similar human tissues, suggesting additional limitations on animal models for translational investigations regarding stiffness-related hypotheses. Taken together, our data support development of tools for evaluation of bowel stiffness in IBD patients for prognostic applications that may enable more accurate prediction of those who will develop fibrosis and more precise prescription of aggressive therapies.

## 1. Introduction

Inflammatory bowel diseases (IBD) are chronic autoinflammatory disorders that can result in Crohn’s disease (CD) or ulcerative colitis (UC). In industrialized countries, the prevalence of IBD continues to increase [1], and there are already approximately 1.6 million Americans living with IBD according to the Crohn’s and Colitis Foundation of America. CD is associated with transmural inflammation in regions throughout the gastrointestinal tract, and approximately 40% to 71% of all CD patients will develop complications requiring surgical resection of the affected bowel segments within 10 years of diagnosis [2–5]. The incidence of bowel strictures needing resection is considerably lower in UC patients, but regions of fibrosis remain a risk factor for colorectal cancer [6–9], leading to surgical resection.

There is a growing interest in developing non-invasive methods to evaluate fibrosis in all IBD patients because new therapies that target fibrosis could help to avoid or delay surgery [10–13]. Clinical evaluation of fibrosis typically requires invasive procedures to isolate patient tissue for histological staining and evaluation of gene expression. Non-invasive procedures such as MRI enterography are beneficial for diagnosis, but only advanced fibrosis can be detected [3,14,15]. Research from other gastrointestinal diseases indicates tissue stiffening may precede fibrosis and act as an early indicator of disease progression [16–19]. Therefore, stiffness monitoring could be used as a non-invasive method for early detection of disease acceleration. Custom methods are needed to evaluate stiffness ex vivo, though, because elastography methods yield relative values with poor spatial resolution that are hard to compare across patients and research models [20]. Direct characterization of human bowel would establish useful baseline values for clinical applications and enable comparisons to potential animal and in vitro models of IBD.

Our goals with this work are (i) to establish baseline mechanical properties for human GI tissue that may inform cell culture substrate design, tissue engineering, and development of prognostic and diagnostic procedures, (ii) to correlate collagen transcription and production with tissue stiffness, and (iii) to directly compare mechanical properties of human tissue to control animal tissue. Animal models of IBD could be a reasonable approach to identifying correlations between fibrosis, stiffness, and clinical symptoms in a controlled manner, and numerous animal models of colitis exist, such as DSS-induced colitis in mice and Johne’s in cattle (see example reviews [21–23]). The porcine gastrointestinal tract is similar to that of humans in terms of architecture, cell type, cell function, and microbiome content, and there are many porcine models of human GI diseases [24,25]. However, these models do not mimic the precise development and progression of fibrosis, strictures, and cancer often associated with IBD, so animal models may not mimic the biomechanics of human disease [26]. Few studies exist to quantify porcine whole-bowel mechanics [27,28] or to compare porcine bowel mechanics to that of human bowel [29]. Similarly, while symptoms of Johne’s Disease in cattle are similar to Crohn’s Disease in humans, the tissue mechanics of bovine small intestine have not been investigated.

Direct comparison of animal tissue to patient tissue also requires specialized equipment. Testing of tissue mechanics in large animals is often performed similar to cadaveric human tissue on standard equipment; however, centimeter-sized samples obtained from human patient surgeries are incompatible with standard testing equipment. Excised GI tissue from mouse models of IBD are also sub-centimeter and incompatible with standard testing equipment. Nano-indentation and atomic force microscopy are useful at characterizing microscale mechanics, but they capture too much heterogeneity to provide distinction across species. To overcome this challenge, we utilized a custom-built multi-scale indenter designed specifically for small excised tissue samples to quantify steady-state modulus [30,31]. In addition to direct comparison across species, this indentation system is compatible with synthetic biomaterials. Biomaterials are often used to mimic diseased tissue, but models of the mechanical microenvironment of IBD are severely limited compared to other well-studied diseases like breast cancer [32–35]. The mechanical microenvironment is an important element of complex diseases [36,37], and in vitro and animal models that mimic the mechanical and biochemical cues of patient bowel could provide new directions for managing and treating IBD.

## 2. Materials and methods

### 2.1 Specimen preparation

**Fig 1:**
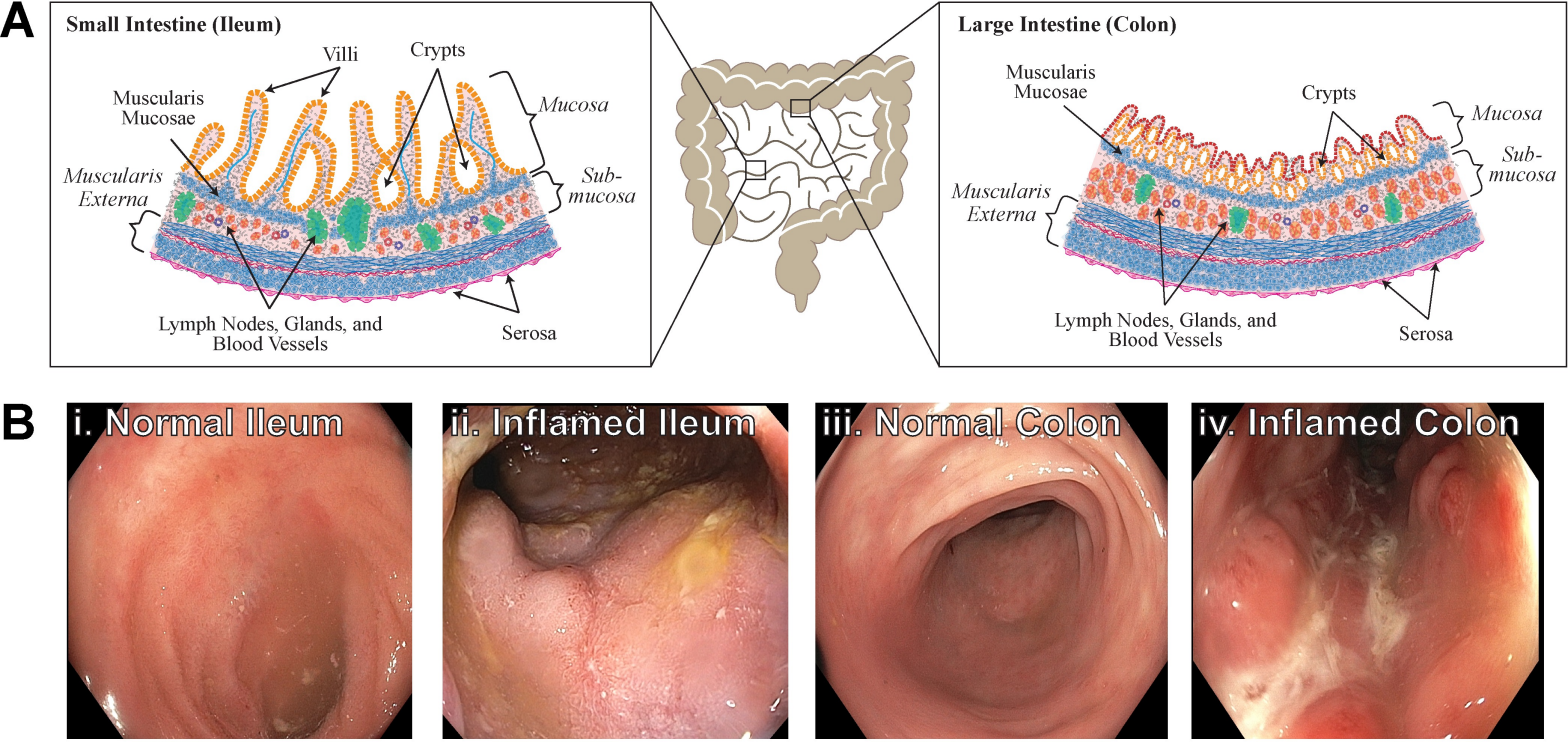
Inflammation affects the behavior of the small and large intestine. (a) Schematic of GI tract in human and representative locations of tissue resected in ileum (left) and colon (right). Normal tissue should be similar in the two regions, though the ileum has long villi extending into the lumen and the colon can have thicker muscular regions for compacting and moving stool. (b) Endoscope images show unaffected ileum (i) and inflamed ileum (ii) regions and unaffected colon (iii) and inflamed colon (iv) are clearly visible by optical inspection.

**Fig 2:**
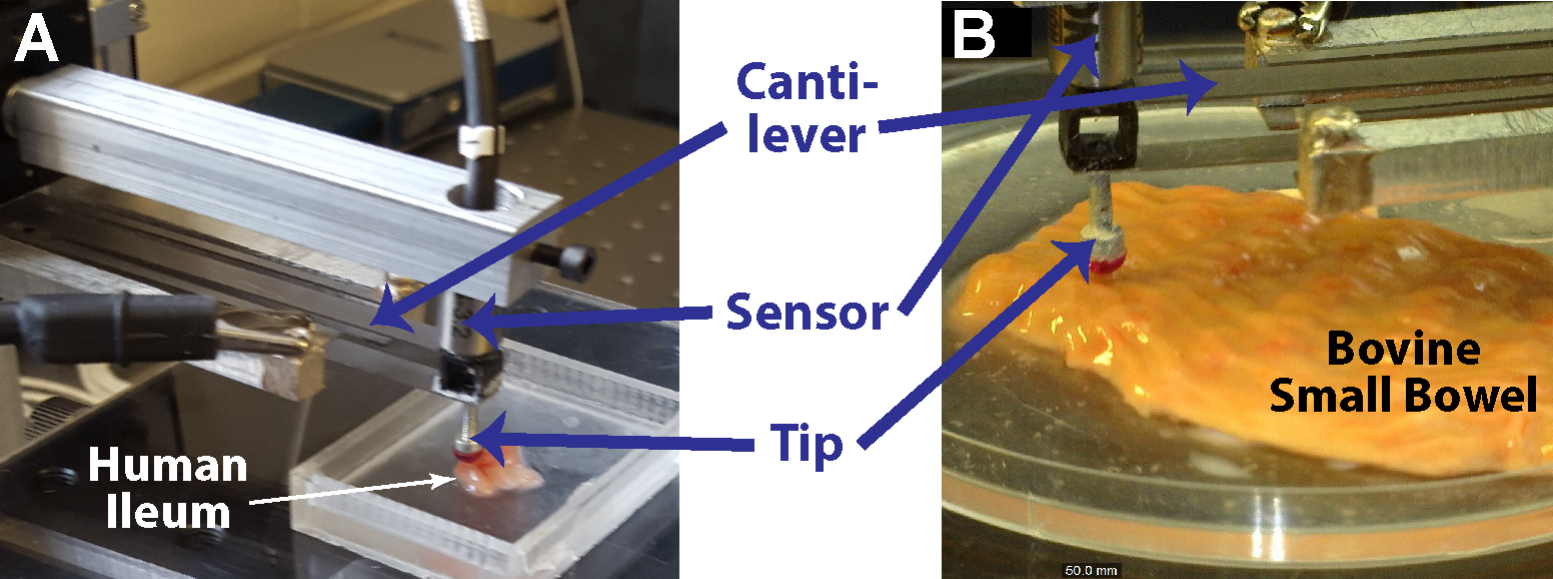
Multi-scale indentation system characterizes mechanical properties of soft tissues. (A) Custom indentation device with sensor positioned with the cantilever in position to test inflamed ileum. (B) Bovine small bowel positioned under the sensor probe with the mucosa exposed. Hydration was maintained by pooling small volumes of saline solution on and around samples (not seen here to improve visualization of tissue).

All human procedures were approved by the Institutional Review Board of the University of Florida (IRB201500440 approved 6/29/2015) which included patient consent, though data was access anonymously for this specific study. Surgically resected portions of bowel were collected from ten CD and UC patients undergoing surgery between February 2016 and November 2016 (S1 Table). Resections yielded sufficient tissue volume for characterization in addition to clinical needs from the ileum only in seven CD patients (3 female and 4 male patients, ages 23–46), colon only in one UC patient (female, age 55), and both the ileum and colon in one UC patient (male, age 33) and one CD patient (male, age 65). Samples were designated “unaffected” or “inflamed” by gross inspection by the pathologist on duty during the given surgery. Sectioned tissue was placed in a cold media solution consisting of Dulbecco’s Modified Eagle Medium (DMEM) and 10% Fetal Bovine Serum (FBS) for subsequent analysis, and samples for mechanical characterization were stored on ice and tested within 4 hours of isolation, similar to previous work characterizing gastrointestinal tissue [30]. Briefly, samples were cut open, rinsed in phosphate buffer saline solution, and placed within a Petri dish. Each sample was positioned so that the mucosa was exposed upward to be indented (Fig 1B). Excised colon samples had 3–10 times more area than ileal samples, which enabled more indentations of both inflamed and unaffected regions. Sections (3–3.5 cm) of colonic tissue from normal 3-month-old C57BL/6 mice were generously donated by Dr. Christian Jobin after euthanasia consistent with American Veterinary Medical Association guidelines. Bovine and porcine tissue was generously donated by Nettle’s Beef and Nettle’s Sausage (Lake City, FL), respectively, harvested under USDA procedures. Sample thickness was approximately 3 mm for human, bovine, and porcine samples and 450 μm for mouse samples. All tissue was prepared and tested at room temperature and kept hydrated with small amounts of DMEM (Fig 2).

### 2.2 Mechanical characterization of freshly-isolated samples

**Fig 3:**
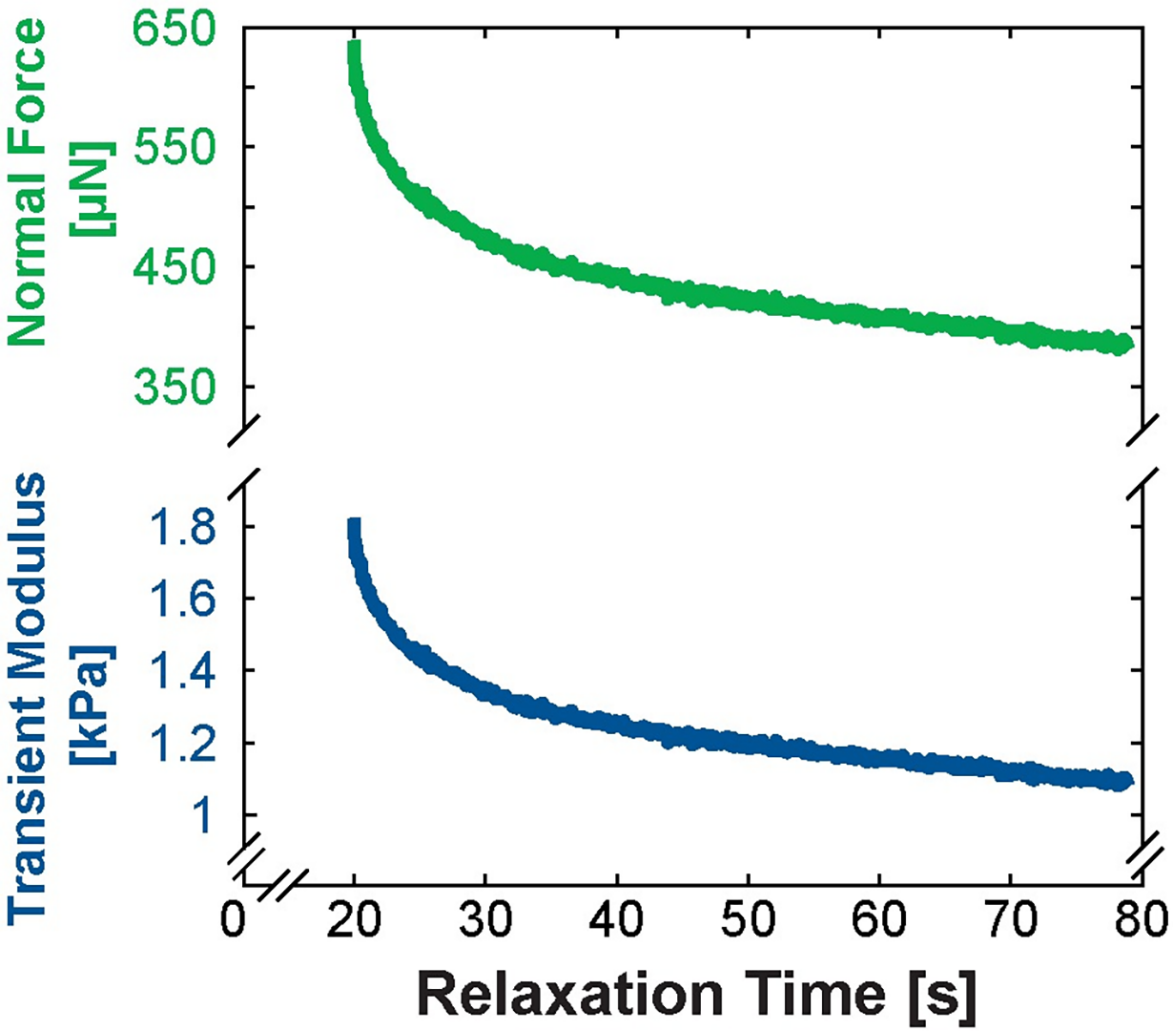
Tissues with time-dependent mechanical properties reach a quasi-steady-state after stress relaxation during indentation. Normal force (top, green line) as a function of relaxation time is used in computing the transient modulus (bottom, blue line) based on a modified Hertz contact model (Equation 1). The Steady-State Modulus (SSM) seen at the end of relaxation is reported throughout this work since SSM reduces strain-rate dependencies in determining the properties of a viscoelastic material.

Human and animal bowel samples were mechanically characterized using a custom-built multi-scale indenter designed for characterizing gastrointestinal tissues and other soft matter (Fig 2) [30,31]. Samples were indented at a rate of 10 μm/s to a depth of 10% of the total thickness of the sample and allowed to relax to a steady-state (Fig 3). An optically polished 4 mm ruby tip was used for indentation of human, porcine, and bovine tissue and a 1 mm glass tip was utilized for murine tissue indentation. Force-displacement data was used to determine a transient modulus based on Hertzian contact mechanics:

$$E_{H}(t)=\frac{3∙F(t)∙(1-\nu^{2})}{4∙\sqrt{R}∙\delta {\left(t\right)}^{\frac{3}{2}}}$$ (1)

where *E*_*H*_*(t)* = transient Hertz modulus; *t* = time; *F(t)* = force as a function of time; *ν* is Poisson’s ratio; *R* is the radius of the indentation tip; and *δ(t)* is the displacement as a function of time. The relaxation portion of the indentation (Fig 3, blue line) was fit to the standard-linear solid model of viscoelasticity to determine a steady-state modulus (SSM):

$$E_{H}(t)=E_{P}+E_{S}e^{-\frac{t}{\tau }}$$ (2)

where *E*_*H*_*(t)* = transient Hertz modulus; *E*_*P*_ = SSM; *E*_*S*_ = strain-rate dependent modulus; *t* = time; and *τ* is the characteristic time, a representation of viscous relaxation, equal to η/E_S_, where η is the viscosity. The limitations of these equations and analyses and additional details on Hertz contact models for soft matter are thoroughly discussed elsewhere [30,31,38].

Human, bovine, and porcine tissue samples were consistently ~3 mm thick, but thin murine tissue required a different indenter tip and deep indentations that were likely affected by the surface below the tissue. To compare murine tissue to others, we performed a separate analysis using the Winkler (elastic foundation) contact model, which is used to model contact of a sphere and a space consisting of a series of elastic columns [39–41]:

$$F(t)=\frac{\pi E_{W}∙R∙{\delta (t)}^{2}}{(1-\nu^{2})∙h}$$ (3)

where *F(t)* = force as a function of time; *t* = time; *E*_*W*_ = Winkler elastic modulus; *R* is the radius of the indentation tip; *δ(t)* is the displacement as a function of time; *ν* is Poisson’s ratio; and *h* is the thickness of the sample [39]. To determine E_W_, force-displacement data during loading was fitted to Equation 3 using the ‘fit’ function in MATLAB (Mathworks) assuming *R* and ν to be constant for all samples and *h* to be constant as measured for each sample.

Though other groups have used ν *=* 0.5 for intestines [42,43], lower values have been used for many other soft tissues [44–47]. We assumed ν *=* 0.4 since uncertainty calculations remain dominated by force and displacement measurements.

### 2.3 Histochemical staining and imaging

Tissue sections were fixed in formalin for 24 hours at 4°C followed by submersion in phosphate buffer solution. Samples were embedded in paraffin, sectioned, and stained by the Molecular Pathology Core at the University of Florida for H&E and Masson’s Trichrome. Images were acquired on a Leica DM 5500B upright microscope under transmitted light. Collagen content of submucosa labeled by Masson’s Trichrome was quantified using ImageJ, and ANOVA was used to assess correlations of age, sex, and collagen content with SSM using JMP Pro 13 (SAS).

### 2.4 RNA isolation, reverse transcription, and quantitative PCR

Total RNA was isolated from surgical small bowel tissues of IBD patients using QIAzol Lysis reagent (Qiagen, Valencia, CA), following the manufacturer’s instructions. The SuperScript® VILO cDNA Synthesis Kit (Thermo Fisher Scientific) was used for cDNA synthesis. PCR was performed using an ABI PRISM StepOnePlus Real time PCR System (Thermo Fisher Scientific) in a 20ul volume containing the cDNA, 2×TaqMan® Gene Expression Master Mix, Nuclease-free water, and 1× Taqman Gene Expression Assay for target genes including CTNNB1, COL4A2, COL1A1, FN, CDH1, MMP1 and TIMP1 (Thermo Fisher Scientific). Reactions were run in triplicate in three independent experiments. The mean of housekeeping gene GAPDH was used as an internal control to normalize target gene expression levels.

### 2.5 Statistical methods

To explore the relationship between steady-state modulus with region and pathology (Section 3.1), we used generalized linear regression with the link of identity and generalized estimating equation (GEE). The link of identity indicates the simple linear relationship between SSM and region/pathology, and GEE is used to account for clustering effect within patients because each patient sample provides unequal numbers of indentations depending on size. For gene expression data (Section 3.2), an unpaired t-test with Welch’s correction was used and graphed in GraphPad Prism 6. For comparison of the total effective modulus and SSM (Section 3.3), statistical significance was determined using a non-parametric Wilcoxon Sum Rank Test with multiple comparisons. Wilcoxon analysis was performed in JMP Pro 13 (SAS software). ANOVA was used to assess correlations of age, sex, and collagen content with SSM, and resulting F-ratios and p-values from analyses are reported. Summary statistics for SSM are reported as mean ± standard deviation in kPa throughout the manuscript.

## 3. Results

### 3.1 Bowel stiffness correlates with inflammation and collagen content

**Fig 4:**
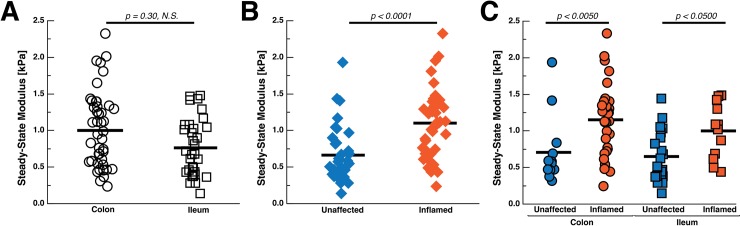
The steady-state modulus of human colon and ileum vary with inflammation state. (A) The difference in stiffness between colon and ileum was not significant (p = 0.30, n = 44 colon measurements on 4 samples from 3 patients and n = 33 ileum measurements on 9 samples from 9 patients). (B) Inflamed regions are stiffer than unaffected regions (p < 0.0001, n = 43 inflamed indentations from 7 patients and n = 34 unaffected indentations from 6 patients). Following this trend, inflamed colonic tissue (n = 31 indentations from 3 patients) is stiffer than unaffected colon regions (n = 13 from 1 patient) and the inflamed ileum (n = 12 from 4 patients) is stiffer than unaffected ileal tissue (n = 21 from 5 patients). Mean stiffness values are represented as a solid black bar.

The difference in stiffness between colon and ileum was not significant (Fig 4A, p = 0.30) but, collectively, inflamed tissue was significantly stiffer than unaffected tissue (Fig 4B, p < 0.0001). Both inflamed colon and inflamed ileum were significantly stiffer than their unaffected counterparts (Fig 4C, p = 0.0024 and p < 0.045, respectively). The inflamed ileum (SSM ± SD = 0.991 ± 0.379 kPa) is stiffer than unaffected regions of the ileum (0.641 ± 0.342 kPa), and inflamed colon (1.143 ± 0.488 kPa) is also stiffer than unaffected regions of the colon (0.698 ± 0.463 kPa). Effective stiffness of bowel did not significantly correlate with age (F Ratio = 1.07, p = 0.32) or sex (F Ratio = 0.0015, p = 0.97) of patients. Mean SSM for each patient was used to determine correlations to avoid weighting analysis in favor of patients with larger tissue resections and thus more indentations.

Collagen content positively correlated with SSM (F Ratio = 3.53, p = 0.093). Calculations utilized mean SSM for each sample and mean collagen content assessed from multiple submucosal regions on a single histological slide. Additional samples would be expected to increase the F ratio and decrease the p value, e.g. including all indentations reduces p-value to <0.0001. Collagen content was not significantly affected by age (F Ratio = 1.34, p = 0.28) but may be influenced marginally by sex (F Ratio = 3.12, p = 0.11). However, consideration of individual indentations (rather than mean per sample) eliminated correlation with sex (F Ratio = 0.004, p = 0.95).

### 3.2 Histology and gene expression are associated with observed mechanical properties

**Fig 5:**
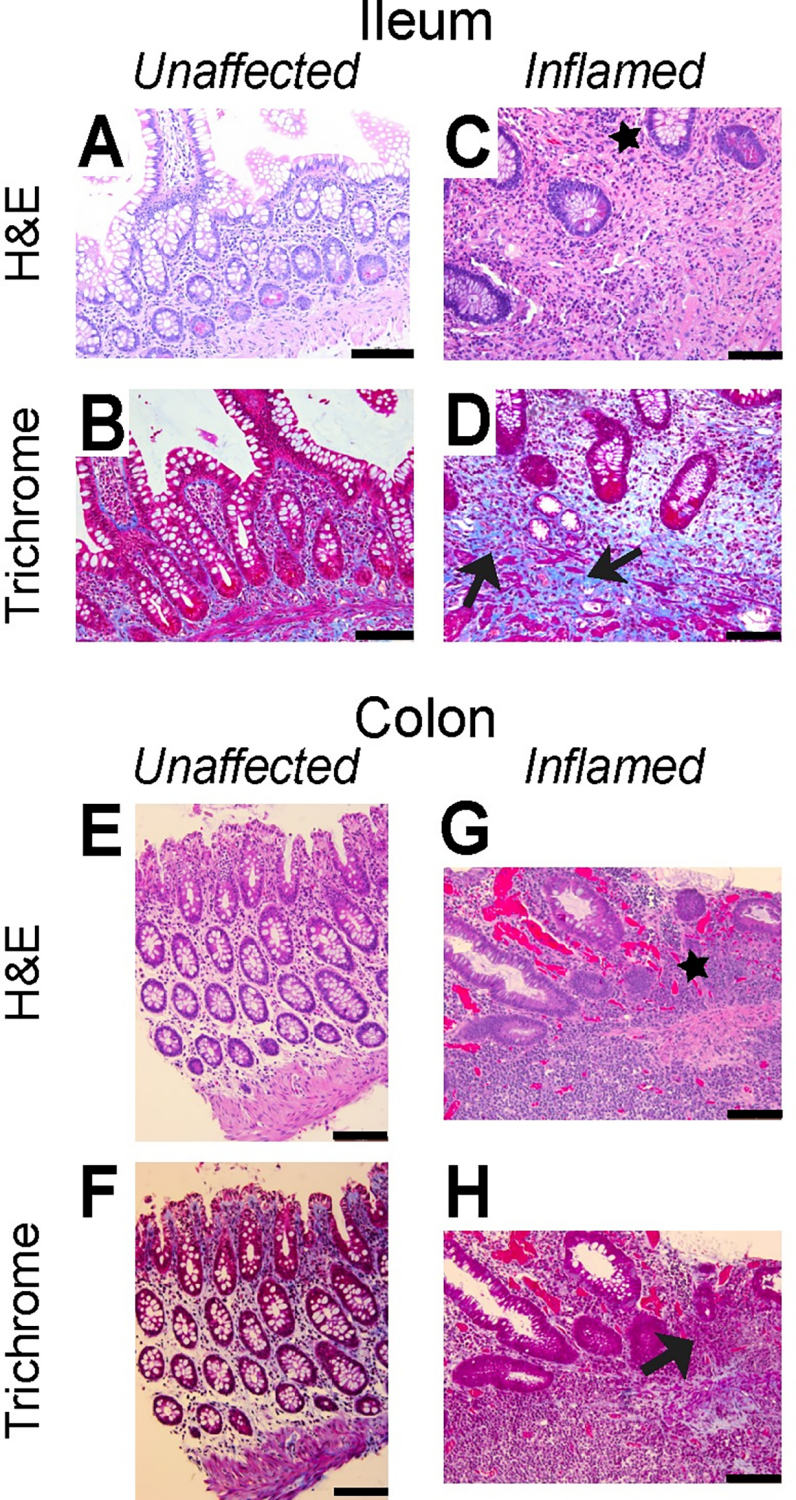
Fibrotic markers are less prevalent in representative staining of the unaffected regions of bowel than inflamed regions. Adjacent regions of Ileum (A-D) and colon (E-H) have been labeled with H&E (A,C,E,G) and Trichrome (B,D,F,H) as indicated. Black arrows highlight collagen clustering while black stars illustrate disrupted mucosal layer in inflamed tissue compared to unaffected tissue segments. Scale bar is 100 μm.

Within inflamed tissue regions, HE staining demonstrates distorted mucosal structure (black stars, Fig 5C and 5G); increased thickness of the mucosa; increased density of infiltrating lymphocytes in the lamina propria; and disorientation of crypt compared to unaffected tissue. Trichrome staining shows increased blue-staining collagen in the inflamed tissue compared to unaffected tissue (black arrows, Fig 5D and 5H).

**Fig 6:**
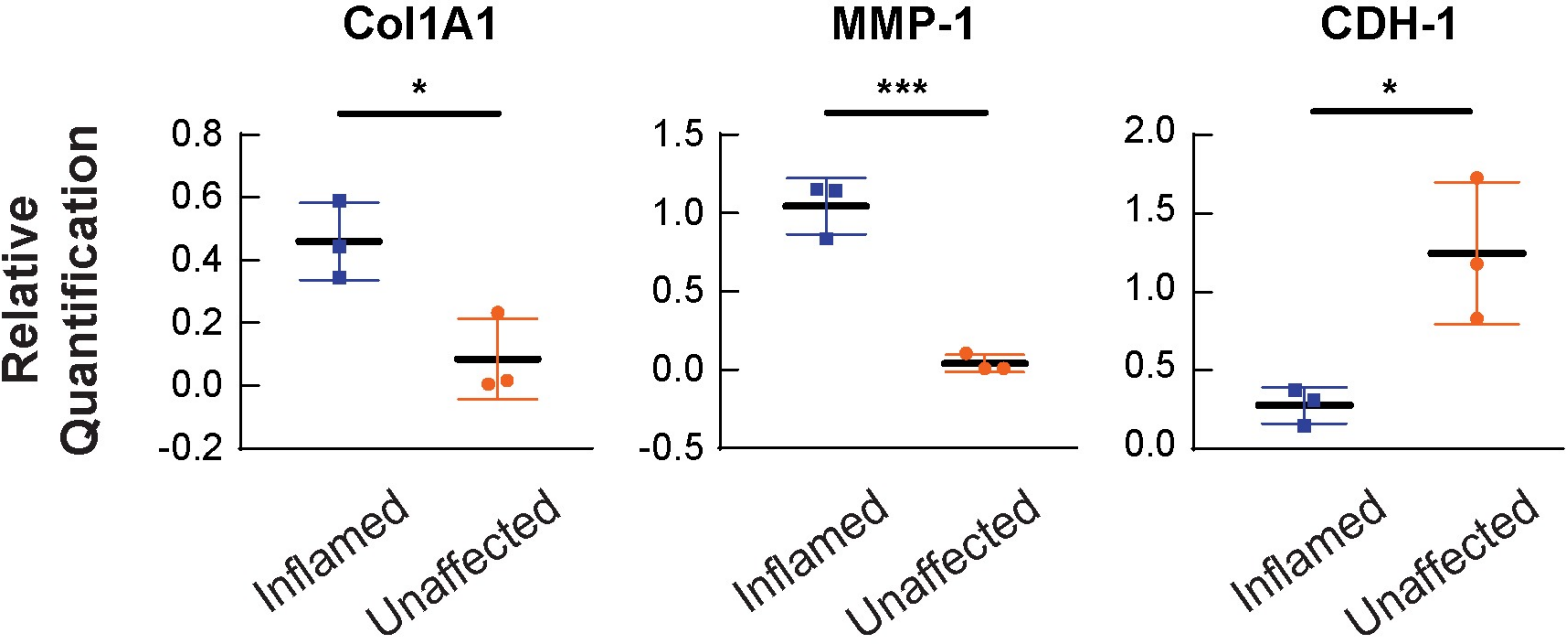
mRNA expression of Col1A1, MMP-1, and CDH-1 is changed in unaffected and inflamed areas of the intestine. mRNA expression profiles of Col1A1 (A), MMP-1 (B), and CDH1 (C) in inflamed and unaffected areas of IBD patients. N = 9 for each group and is represented as the average of 3 separate experiments. Collagen type I is significantly upregulated in the inflamed areas (*p = 0.0110) along with MMP-1 expression upregulation in inflamed areas (***p = 0.0032). The expression of CDH1, E-cadherin, is downregulated in inflamed areas (*p = 0.0288). Gene expression that resulted in no significant change is detailed in S1 Fig.

Consistent with histological findings, expression of collagen type I was upregulated in the inflamed cases compared to the unaffected cases (p = 0.011, Fig 6) and increased collagen content in inflamed samples compared to unaffected was confirmed by analysis of collagen content by image processing (p = 0.044). We observe no significant changes in the expression levels of collagen IV, the primary component of the basement membrane for intestinal epithelial cells. The expression level of the matrix-remodeling matrix metalloproteinase 1 (MMP1) is upregulated in the inflamed cases compared to the unaffected cases (p = 0.0032, Fig 6). There is not an observable change in the expression levels of tissue inhibitor of metalloproteinase 1 (TIMP-1) nor fibronectin. E-cadherin is down-regulated in inflamed tissue (p = 0.0288, Fig 6), and we observe no significant change in the expression of β-catenin within our cohort (S1 Fig).

### 3.3 Bowel histology and stiffness in control animals does not mimic unaffected regions of human tissue

**Fig 7:**
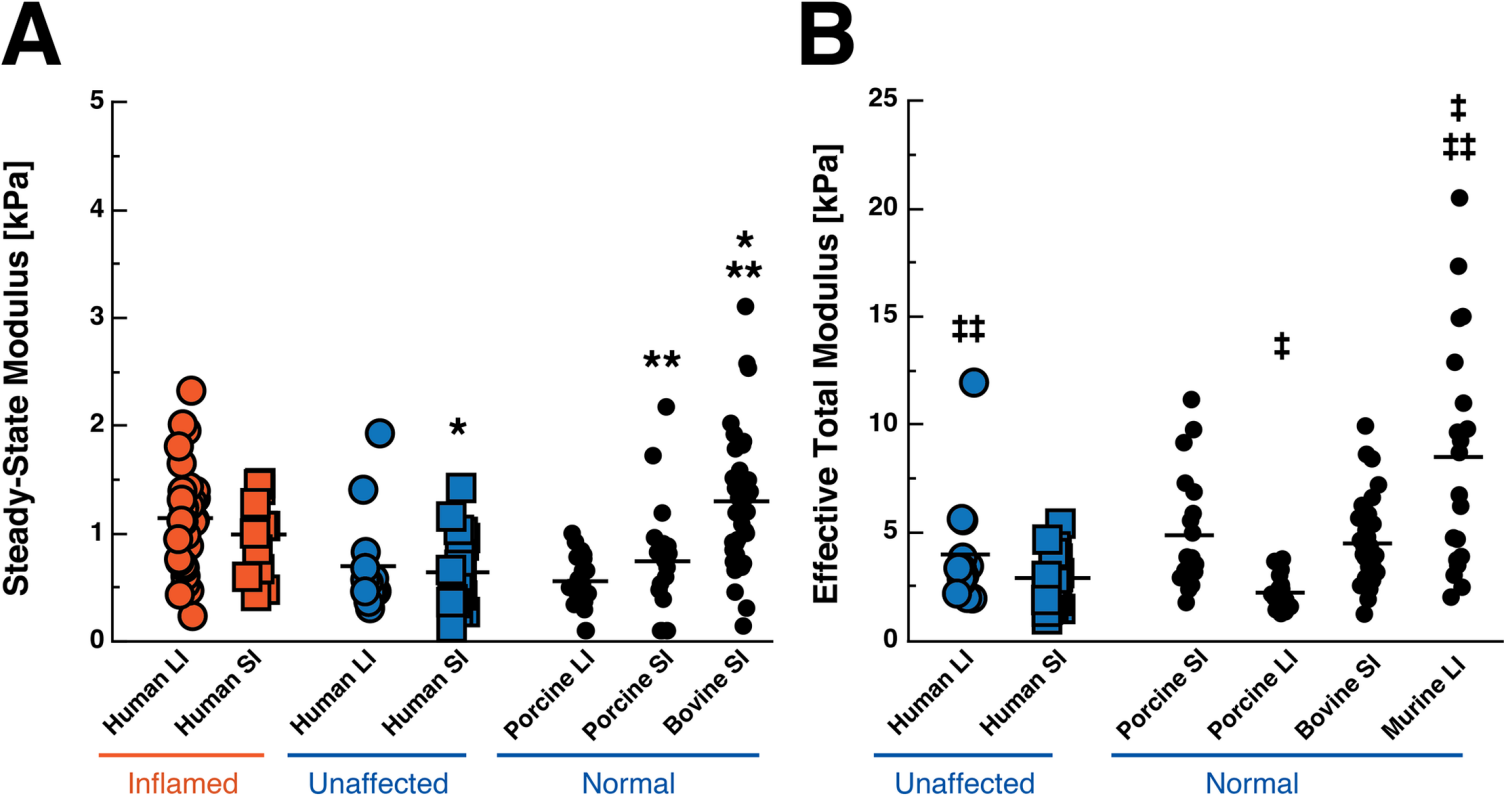
Comparison of the steady-state modulus (SSM) and effective total modulus of human tissue and control animal tissue. Inflamed human tissue is displayed in orange while unaffected human and normal animal tissue is displayed in blue. (a) The SSM is representative of the steady-state behavior of a viscoelastic material after stress relaxation (Eqs 1 and 2). (b) The effective total modulus is determined from using the Winkler contact model which assumes that the tissue is a foundation of purely elastic springs (Eq 3). Group means are displayed as a solid black line within each group. Each dot represents one indentation. SI = small intestine, LI = large intestine. * p < 0.0001, ** p = 0.0004, ‡ p<0.0001, ‡‡ p = 0.0043. Note: Scales of y-axes are different for each graph as effective total modulus yields higher values than SSM.

The SSM of some unaffected animal models is different than that of the unaffected areas of our human patients (Fig 7A). For example, bovine small bowel segments (1.299 ± 0.615 kPa) are nearly twice as stiff as unaffected human small bowel (0.641 ± 0.342, p < 0.0001), though porcine small bowel tissue (0.743 ± 0.525 kPa) is similar to human small bowel samples (p = 0.611); porcine small bowel is also softer than bovine small bowel (p = 0.0004). In addition, porcine colonic tissue (0.5583 ± 0.2404 kPa) also has a similar SSM to human colonic tissue (0.698 ± 0.463, p = 0.7200).

Because of the thin nature of murine colon samples, force-displacement data from unaffected human and animal samples were fitted to the Winkler contact model (see Materials and Methods section). Similar trends to SSM (Fig 7A) were observed in the effective total modulus values from the Winkler model (Fig 7B). However, murine colon samples (8.493 ± 5.365 kPa, Fig 7B) were significantly stiffer than the unaffected human (3.985 ± 2.656 kPa, p = 0.0043) and normal porcine large intestine (2.221 ± 0.700 kPa, p < 0.0001) samples.

**Fig 8:**
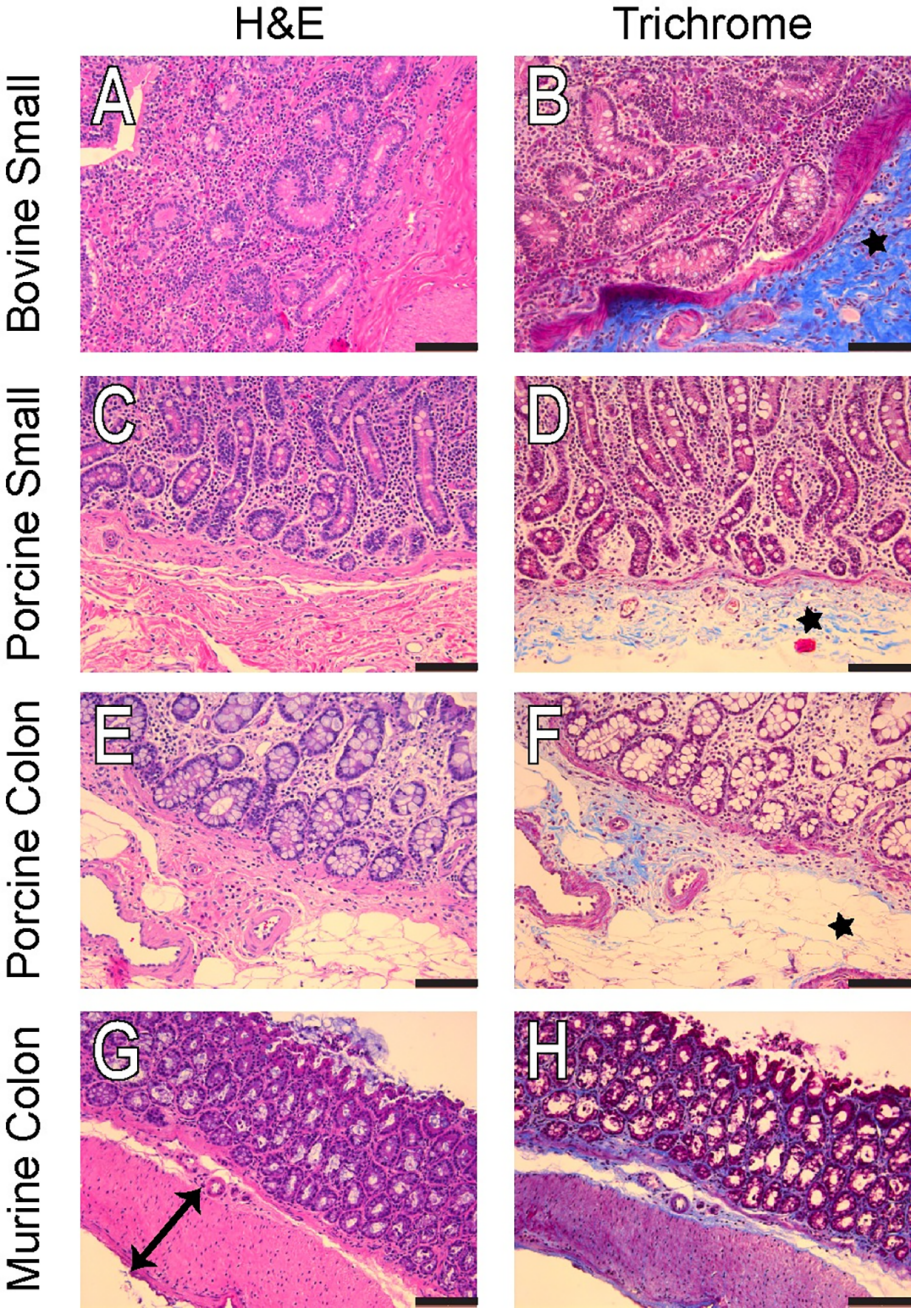
**Representative H&E and Masson’s Trichrome of the cow small bowel (A,B), pig small bowel (C,D), pig colon (E,F), and mouse colon (G,H).** The mouse colon has a very distinct muscle layer highlighted by the black arrow (G), while the cow small bowel has a very large area of collagen deposition (B, black star) that is absent in the pig small bowel and colon (black stars, D and F). Scale bar is 100 μm.

Histology staining shows distinct intestinal mucosa architecture and differential collagen deposition pattern among bovine, porcine and murine tissue (Fig 8A, 8C, 8E and 8G). Consistent with modulus data, bovine small bowel tissue contains relatively more collagen fiber compared to porcine gut and murine colon (Fig 8B, 8D, 8F and 8H).

## 4. Discussion

Our direct characterization has established that inflamed regions of human bowel are significantly stiffer than uninflamed regions and has confirmed the general trends observed previously via compression [48] and ultrasound elastography [15,49–52]. Clinical elastography measurements may prove useful for diagnostic, prognostic, and surgical planning applications; however, because of the large variation in absolute values and nomenclature in mechanical characterization of IBD, our methods for direct comparison and analysis are essential.

We found that collagen content correlates with increased stiffness in bowel, as suggested by other qualitative data [49], but there are many contributors to increased tissue stiffness. We find that expression of collagen I subunit ColA1 is in fact upregulated, but collagen fibril formation and assembly involves numerous additional genes that we did not examine here [50]. In addition, individual ECM gene and protein expression do not necessarily correlate with stiffness because of the wide range of contributing genes [51]. We found upregulation of matrix metalloproteinase 1 (MMP-1) but no significant change in collagen IV, tissue inhibitor of metalloproteinase 1 (TIMP-1), nor fibronectin. The balance between MMP and TIMP activity is critical in regulating ECM remodeling [52], and a change in MMP with no change in TIMP could reflect the imbalance in matrix deposition seen in histology.

We also observed changes in cell-cell contacts associated with the inner mucosal layer of the gut, and the disrupted mucosal layer may contribute to dysregulation of matrix remodeling and affect stiffness through alterations in the cells themselves. We found E-cadherin to be significantly downregulated in the inflamed bowel, which may be affecting enzymatic remodeling of ECM [53]. Our gene expression data suggest, as in other GI diseases, global imbalances in matrix production are related to changes in stiffness, but the contribution of specific subtypes of matrix proteins and modifiers demands further investigation.

Increased cross-linking of matrix could also be contributing to increased stiffness seen in inflamed patient tissue[19,54,55]. Cross-linking, e.g. lysyl oxidase-mediated, was not examined in our initial characterization of gene expression in patient tissue, but animal data suggest collagen cross-linking plays an important role in GI tissue stiffness [16,56]. Future investigations interested in biophysical aspects of IBD and comparisons of patient tissue to animal models should consider collagen crosslinking and ensure similarities across species and etiologies.

By using our custom equipment compatible with excised patient samples and animal tissue, we have also determined that effective stiffness of normal bovine bowel does not match effective stiffness of normal human or porcine bowel tested. Because it is possible that substrate effects from silicone underneath the thin samples affect measurements, we used a separate analysis (Winkler elastic foundation contact model) to compare murine intestines to other species.

Though mice are often used to model IBD [21,57], we found that murine colon is significantly stiffer than human colon tested, which could limit the translatability of findings with a significant mechanobiology component. Epithelial cell behavior is heavily influenced by the mechanical microenvironment, for example in epithelial-to-mesenchymal transition [33,58,59], ECM deposition[60], and cell division and migration [61–64], so stiffer submucosal layers in animals may contribute to epithelial (dys)function differently than softer regions in humans. Since porcine bowel is similar to that of human in physiology, architecture, and stiffness, GI tissue mechanics and mechanobiology studies in pigs may translate more readily to humans.

We have demonstrated a strong relationship between intestinal stiffness and human disease, but the effect of stiffness on progression of IBD warrants further study. Investigating the impact of mechanical properties of the gut on resident cells, immune cells, and microbiota will require models of disease progression that mimic human tissue mechanics. While this may be possible in certain animal models, it is worth careful consideration of the mechanical microenvironment and its potential effect on outcomes once translated to humans, given the dissimilar stiffness articulated by our findings. Stiffness-mimicking in vitro models that accommodate human cells in biomimetic configurations will likely prove valuable complements to animal models in mechanistic studies of fibrosis in IBD. In vitro models have already been used to demonstrate that substrate stiffness affects GI cell stromal cell activation [48], epithelial function [65–67], and epithelial-to-mesenchymal transition and cancer-like progression [68–71]. A wide range of in vitro and in vivo models should be leveraged to explore stiffness-related mechanisms of human IBD in the future.

## Supporting information

S1 Fig Gene expression data for collagen type IV, β-catenin, fibronectin, and TIMP-1. N = 9 per group and is represented as the average of 3 separate experiments. We did not detect significant changes in these genes within our cohort.
(TIF)

S1 Table Patient characteristics for obtained tissue samples. M = Male, F = Female, CD = Crohn’s Disease, UC = Ulcerative Colitis.
(DOCX)

S2 Table Raw data used to construct figures. CD = Crohn’s Disease, UC = Ulcerative Colitis.
(XLSX)

## References

[1] Kappelman MD, Moore KR, Allen JK, Cook SF. Recent Trends in the Prevalence of Crohn’s Disease and Ulcerative Colitis in a Commercially Insured US Population. Dig Dis Sci. Springer US; 2013;58: 519–525. doi: 10.1007/s10620-012-2371-5 22926499 10.1007/s10620-012-2371-5 PMC3576554

[2] Rieder F, Zimmermann EM, Remzi FH, Sandborn WJ. Crohn's disease complicated by strictures: a systematic review. Gut. BMJ Publishing Group; 2013;62: 1072–1084. doi: 10.1136/gutjnl-2012-304353 23626373 10.1136/gutjnl-2012-304353 PMC4884453

[3] Rieder F, Fiocchi C. Intestinal fibrosis in IBD—a dynamic, multifactorial process. Nat Rev Gastroenterol Hepatol. Nature Publishing Group; 2009;6: 228–235. doi: 10.1038/nrgastro.2009.31 19347014 10.1038/nrgastro.2009.31

[4] Rieder F, Brenmoehl J, Leeb S, Schölmerich J, Rogler G. Wound healing and fibrosis in intestinal disease. Gut. BMJ Publishing Group; 2007;56: 130–139. doi: 10.1136/gut.2006.090456 17172588 10.1136/gut.2006.090456 PMC1856649

[5] Fiocchi C, Lund PK. Themes in fibrosis and gastrointestinal inflammation. Am J Physiol Gastrointest Liver Physiol. 2011;300: G677–83. doi: 10.1152/ajpgi.00104.2011 21415411 10.1152/ajpgi.00104.2011 PMC3094134

[6] Edwards FC, Truelove SC. THE COURSE AND PROGNOSIS OF ULCERATIVE COLITIS. III. COMPLICATIONS. Gut. BMJ Publishing Group; 1964;5: 1–22. 14127503 10.1136/gut.5.1.1 PMC1552214

[7] De Dombal FT, Watts JM, Watkinson G, Goligher JC. Local complications of ulcerative colitis: stricture, pseudopolyposis, and carcinoma of colon and rectum. Br Med J. BMJ Publishing Group; 1966;1: 1442–1447. 5933046 10.1136/bmj.1.5501.1442 PMC1844640

[8] Gumaste V, Sachar DB, Greenstein AJ. Benign and malignant colorectal strictures in ulcerative colitis. Gut. BMJ Publishing Group; 1992;33: 938–941. 1644333 10.1136/gut.33.7.938 PMC1379408

[9] de Bruyn JR, Meijer SL, Wildenberg ME, Bemelman WA, van den Brink GR, D'Haens GR. Development of Fibrosis in Acute and Longstanding Ulcerative Colitis. J Crohns Colitis. 2015;9: 966–972. doi: 10.1093/ecco-jcc/jjv133 26245217 10.1093/ecco-jcc/jjv133

[10] Salari-Sharif P, Abdollahi M. Phosphodiesterase 4 inhibitors in inflammatory bowel disease: a comprehensive review. Curr Pharm Des. 2010;16: 3661–3667. 21128899 10.2174/138161210794079209

[11] Yamagata M, Mikami T, Tsuruta T, Yokoyama K, Sada M, Kobayashi K, et al Submucosal fibrosis and basic-fibroblast growth factor-positive neutrophils correlate with colonic stenosis in cases of ulcerative colitis. Digestion. Karger Publishers; 2011;84: 12–21. doi: 10.1159/000320773 21304240 10.1159/000320773

[12] Pellino G, Pallante P, Selvaggi F. Novel biomarkers of fibrosis in Crohn's disease. World J Gastrointest Pathophysiol. 2016;7: 266–275. doi: 10.4291/wjgp.v7.i3.266 27574564 10.4291/wjgp.v7.i3.266 PMC4981766

[13] Speca S, Rousseaux C, Dubuquoy C, Rieder F, Vetuschi A, Sferra R, et al Novel PPARγ Modulator GED-0507-34 Levo Ameliorates Inflammation-driven Intestinal Fibrosis. Inflammatory bowel diseases. 2016;22: 279–292. doi: 10.1097/MIB.0000000000000618 26535766 10.1097/MIB.0000000000000618 PMC4718865

[14] Wynn TA. Cellular and molecular mechanisms of fibrosis Altmann DM, Douek DC, editors. The Journal of Pathology. John Wiley & Sons, Ltd; 2008;214: 199–210. 18161745 10.1002/path.2277 PMC2693329

[15] Adler J, Stidham RW, Higgins PDR. Bringing the Inflamed and Fibrotic Bowel into Focus: Imaging in Inflammatory Bowel Disease. Gastroenterology & Hepatology. Millenium Medical Publishing; 2009;5: 705. PMC2886363 37967416

[16] Georges PC, Hui J-J, Gombos Z, McCormick ME, Wang AY, Uemura M, et al Increased stiffness of the rat liver precedes matrix deposition: implications for fibrosis. Am J Physiol Gastrointest Liver Physiol. 2007;293: G1147–54. doi: 10.1152/ajpgi.00032.2007 17932231 10.1152/ajpgi.00032.2007

[17] Degos F, Perez P, Roche B, Mahmoudi A, Asselineau J, Voitot H, et al Diagnostic accuracy of FibroScan and comparison to liver fibrosis biomarkers in chronic viral hepatitis: A multicenter prospective study (the FIBROSTIC study). Journal of Hepatology. 2010;53: 1013–1021. doi: 10.1016/j.jhep.2010.05.035 20850886 10.1016/j.jhep.2010.05.035

[18] Perepelyuk M, Terajima M, Wang AY, Georges PC, Janmey PA, Yamauchi M, et al Hepatic stellate cells and portal fibroblasts are the major cellular sources of collagens and lysyl oxidases in normal liver and early after injury. Am J Physiol Gastrointest Liver Physiol. American Physiological Society; 2013;304: G605–14. doi: 10.1152/ajpgi.00222.2012 23328207 10.1152/ajpgi.00222.2012 PMC3602686

[19] Wells RG. Tissue mechanics and fibrosis. 2013;1832: 884–890. doi: 10.1016/j.bbadis.2013.02.007 23434892 10.1016/j.bbadis.2013.02.007 PMC3641165

[20] Stidham RW, Xu J, Johnson LA, Kim K, Moons DS, McKenna BJ, et al Ultrasound Elasticity Imaging for Detecting Intestinal Fibrosis and Inflammation in Rats and Humans With Crohn's Disease. Gastroenterology. 2011;141: 819–826.e1. doi: 10.1053/j.gastro.2011.07.027 21784048 10.1053/j.gastro.2011.07.027 PMC4934420

[21] Wirtz S, Neurath MF. Mouse models of inflammatory bowel disease. Advanced Drug Delivery Reviews. 2007;59: 1073–1083. doi: 10.1016/j.addr.2007.07.003 17825455 10.1016/j.addr.2007.07.003

[22] Davis WC, Kuenstner JT, Singh SV. Resolution of Crohn“s (Johne”s) disease with antibiotics: what are the next steps? Expert Rev Gastroenterol Hepatol. Taylor & Francis; 2017;11: 393–396. doi: 10.1080/17474124.2017.1300529 28276276 10.1080/17474124.2017.1300529

[23] Gadaleta RM, Garcia-Irigoyen O, Moschetta A. Exploration of Inflammatory Bowel Disease in Mice: Chemically Induced Murine Models of Inflammatory Bowel Disease (IBD). Hoboken, NJ, USA: John Wiley & Sons, Inc; 2011 pp. 13–28. 10.1002/cpmo.20 28252200

[24] Aigner B, Renner S, Kessler B, Klymiuk N, Kurome M, Wünsch A, et al Transgenic pigs as models for translational biomedical research. J Mol Med. Springer-Verlag; 2010;88: 653–664. doi: 10.1007/s00109-010-0610-9 20339830 10.1007/s00109-010-0610-9

[25] Ziegler A, Gonzalez L, Blikslager A. Large Animal Models: The Key to Translational Discovery in Digestive Disease Research. Cellular and Molecular Gastroenterology and Hepatology. 2016;2: 716–724. doi: 10.1016/j.jcmgh.2016.09.003 28090566 10.1016/j.jcmgh.2016.09.003 PMC5235339

[26] Seok J, Warren HS, Cuenca AG, Mindrinos MN, Baker HV, Xu W, et al Genomic responses in mouse models poorly mimic human inflammatory diseases. Proc Natl Acad Sci USA. National Acad Sciences; 2013;110: 3507–3512. doi: 10.1073/pnas.1222878110 23401516 10.1073/pnas.1222878110 PMC3587220

[27] Qiao Y, Pan E, Chakravarthula SS, Han F, Liang J, Gudlavalleti S. Measurement of mechanical properties of rectal wall. J Mater Sci Mater Med. Kluwer Academic Publishers; 2005;16: 183–188. doi: 10.1007/s10856-005-5988-5 15744608 10.1007/s10856-005-5988-5

[28] Carniel EL, Gramigna V, Fontanella CG, Frigo A, Stefanini C, Rubini A, et al Characterization of the anisotropic mechanical behaviour of colonic tissues: experimental activity and constitutive formulation. Exp Physiol. 2014;99: 759–771. doi: 10.1113/expphysiol.2013.076091 24486449 10.1113/expphysiol.2013.076091

[29] Christensen MB, Oberg K, Wolchok JC. Tensile properties of the rectal and sigmoid colon: a comparative analysis of human and porcine tissue. Springerplus. Springer; 2015;4: 142 doi: 10.1186/s40064-015-0922-x 25977885 10.1186/s40064-015-0922-x PMC4414857

[30] Stewart DC, Rubiano A, Santisteban MM, Shenoy V, Qi Y, Pepine CJ, et al Hypertension-linked mechanical changes of rat gut. Acta Biomater. 2016;45: 296–302. doi: 10.1016/j.actbio.2016.08.045 27567964 10.1016/j.actbio.2016.08.045 PMC5069177

[31] Stewart DC, Rubiano A, Dyson K, Simmons CS. Mechanical characterization of human brain tumors from patients and comparison to potential surgical phantoms. Engler AJ, editor. PLoS ONE. 2017;12: e0177561 doi: 10.1371/journal.pone.0177561 28582392 10.1371/journal.pone.0177561 PMC5459328

[32] National Academy of Engineering. Frontiers of Engineering. Washington, D.C.: National Academies Press; 2017 doi: 10.17226/23659

[33] Wei SC, Fattet L, Tsai JH, Guo Y, Pai VH, Majeski HE, et al Matrix stiffness drives epithelial-mesenchymal transition and tumour metastasis through a TWIST1-G3BP2 mechanotransduction pathway. Nat Cell Biol. Nature Research; 2015;17: 678–688. doi: 10.1038/ncb3157 25893917 10.1038/ncb3157 PMC4452027

[34] Levental KR, Yu H, Kass L, Lakins JN, Egeblad M, Erler JT, et al Matrix Crosslinking Forces Tumor Progression by Enhancing Integrin Signaling. Cell. 2009;139: 891–906. doi: 10.1016/j.cell.2009.10.027 19931152 10.1016/j.cell.2009.10.027 PMC2788004

[35] Vidi P-A, Bissell MJ, Lelièvre SA. Three-Dimensional Culture of Human Breast Epithelial Cells: The How and the Why. Totowa, NJ: Humana Press; 2012 pp. 193–219. 10.1007/978-1-62703-125-7_13 PMC3666567 23097109

[36] Levental I, Georges PC, Janmey PA. Soft biological materials and their impact on cell function. Soft Matter. 2007. 10.1039/b610522j 32900146

[37] Huynh J, Nishimura N, Rana K, Peloquin JM, Califano JP, Montague CR, et al Age-Related Intimal Stiffening Enhances Endothelial Permeability and Leukocyte Transmigration. Science Translational Medicine. American Association for the Advancement of Science; 2011;3: 112–122. doi: 10.1126/scitranslmed.3002761 22158860 10.1126/scitranslmed.3002761 PMC3693751

[38] Bush BG, Shapiro JM, DelRio FW, Cook RF, Oyen ML. Mechanical measurements of heterogeneity and length scale effects in PEG-based hydrogels. Soft Matter. 2015;11: 7191–7200. doi: 10.1039/c5sm01210d 26255839 10.1039/c5sm01210d PMC4571184

[39] Johnson KL. Contact Mechanics. 1st ed. Cambridge, United Kingdom: Cambridge University Press; 1985.

[40] Põdra P, Andersson S. Wear simulation with the Winkler surface model. Wear. 1997;207: 79–85. doi: 10.1016/S0043-1648(96)07468-6

[41] Di Paola M, Marino F, Zingales M. A generalized model of elastic foundation based on long-range interactions: Integral and fractional model. International Journal of Solids and Structures. Elsevier Ltd; 2009;46: 3124–3137. doi: 10.1016/j.ijsolstr.2009.03.024

[42] Hoeg HD, Slatkin AB, Burdick JW, Grundfest WS. Biomechanical modeling of the small intestine as required for the design and operation of a robotic endoscope. ROBOT-00. IEEE; 2000;2: 1599–1606 vol.2. doi: 10.1109/ROBOT.2000.844825

[43] Zhang C, Liu H, Tan R, Li H. Modeling of Velocity-dependent Frictional Resistance of a Capsule Robot Inside an Intestine Tribol Lett. 1st ed. Springer US; 2012;47: 295–301. doi: 10.1007/s11249-012-9980-1

[44] Armitage OE, Oyen ML. Indentation across interfaces between stiff and compliant tissues. Acta Biomater. 2017;56: 36–43. doi: 10.1016/j.actbio.2016.12.036 28062353 10.1016/j.actbio.2016.12.036

[45] Darling EM, Zauscher S, Block JA, Guilak F. A thin-layer model for viscoelastic, stress-relaxation testing of cells using atomic force microscopy: do cell properties reflect metastatic potential? Biophys J. 2007;92: 1784–1791. doi: 10.1529/biophysj.106.083097 17158567 10.1529/biophysj.106.083097 PMC1796808

[46] Meliga SC, Coffey JW, Crichton ML, Flaim C, Veidt M, Kendall MAF. The hyperelastic and failure behaviors of skin in relation to the dynamic application of microscopic penetrators in a murine model. Acta Biomater. 2016;48: 1–16. doi: 10.1016/j.actbio.2016.10.021 27746361 10.1016/j.actbio.2016.10.021

[47] Kyriacou SK, Mohamed A, Miller K, Neff S. Brain mechanics For neurosurgery: modeling issues. Biomech Model Mechanobiol. Springer-Verlag; 2002;1: 151–164. doi: 10.1007/s10237-002-0013-0 14595547 10.1007/s10237-002-0013-0

[48] Johnson LA, Rodansky ES, Sauder KL, Horowitz JC, Mih JD, Tschumperlin DJ, et al Matrix stiffness corresponding to strictured bowel induces a fibrogenic response in human colonic fibroblasts. Inflammatory bowel diseases. NIH Public Access; 2013;19: 891–903. doi: 10.1097/MIB.0b013e3182813297 23502354 10.1097/MIB.0b013e3182813297 PMC3766259

[49] Swift J, Ivanovska IL, Buxboim A, Harada T, Dingal PCDP, Pinter J, et al Nuclear Lamin-A Scales with Tissue Stiffness and Enhances Matrix-Directed Differentiation. Science. American Association for the Advancement of Science; 2013;341: 1240104–1240104. doi: 10.1126/science.1240104 23990565 10.1126/science.1240104 PMC3976548

[50] Birk DE, Brückner P. Collagens, Suprastructures, and Collagen Fibril Assembly The Extracellular Matrix: an Overview. Berlin, Heidelberg: Springer Berlin Heidelberg; 2010 pp. 77–115. doi: 10.1007/978-3-642-16555-9_3

[51] Smith LR, Barton ER. Collagen content does not alter the passive mechanical properties of fibrotic skeletal muscle in mdx mice. Am J Physiol, Cell Physiol. American Physiological Society; 2014;306: C889–C898. doi: 10.1152/ajpcell.00383.2013 24598364 10.1152/ajpcell.00383.2013 PMC4024713

[52] Nagase H, Visse R, Murphy G. Structure and function of matrix metalloproteinases and TIMPs. Cardiovascular Research. 2006;69: 562–573. doi: 10.1016/j.cardiores.2005.12.002 16405877 10.1016/j.cardiores.2005.12.002

[53] Sengupta N, MacDonald TT. The role of matrix metalloproteinases in stromal/epithelial interactions in the gut. Physiology. American Physiological Society; 2007;22: 401–409. doi: 10.1152/physiol.00027.2007 18073413 10.1152/physiol.00027.2007

[54] Elbjeirami WM, Yonter EO, Starcher BC, West JL. Enhancing mechanical properties of tissue-engineered constructs via lysyl oxidase crosslinking activity. J Biomed Mater Res. Wiley Subscription Services, Inc., A Wiley Company; 2003;66A: 513–521. doi: 10.1002/jbm.a.10021 12918034 10.1002/jbm.a.10021

[55] Huber A, Ebner L, Heverhagen JT, Christe A. State-of-the-art imaging of liver fibrosis and cirrhosis: A comprehensive review of current applications and future perspectives. European Journal of Radiology Open. 2015;2: 90–100. doi: 10.1016/j.ejro.2015.05.002 26937441 10.1016/j.ejro.2015.05.002 PMC4750581

[56] Baker A-M, Bird D, Lang G, Cox TR, Erler JT. Lysyl oxidase enzymatic function increases stiffness to drive colorectal cancer progression through FAK. Oncogene. Nature Publishing Group; 2013;32: 1863–1868. doi: 10.1038/onc.2012.202 22641216 10.1038/onc.2012.202

[57] Mizoguchi A, Takeuchi T, Himuro H, Okada T, Mizoguchi E. Genetically engineered mouse models for studying inflammatory bowel disease. Arends MJ, White ES, Whitelaw CBA, editors. The Journal of Pathology. John Wiley & Sons, Ltd; 2016;238: 205–219. doi: 10.1002/path.4640 26387641 10.1002/path.4640 PMC4689626

[58] Leight JL, Wozniak MA, Chen S, Lynch ML, Chen CS. Matrix rigidity regulates a switch between TGF-β1-induced apoptosis and epithelial-mesenchymal transition. Wang Y-L, editor. Mol Biol Cell. 2012;23: 781–791. doi: 10.1091/mbc.E11-06-0537 22238361 10.1091/mbc.E11-06-0537 PMC3290638

[59] Matsuzaki S, Darcha C, Pouly J-L, Canis M. Effects of matrix stiffness on epithelial to mesenchymal transition-like processes of endometrial epithelial cells: Implications for the pathogenesis of endometriosis. Sci Rep. Nature Publishing Group; 2017;7: 44616 doi: 10.1038/srep44616 28303918 10.1038/srep44616 PMC5356009

[60] Jones J. Substrate stiffness regulates extracellular matrix deposition by alveolar epithelial cells. Research and Reports in Biology. Dove Press; 2011;2: 1–12. doi: 10.2147/RRB.S13178 23204878 10.2147/RRB.S13178 PMC3510703

[61] Das T, Safferling K, Rausch S, Grabe N, Boehm H, Spatz JP. A molecular mechanotransduction pathway regulates collective migration of epithelial cells. Nat Cell Biol. 2015;17: 276–287. doi: 10.1038/ncb3115 25706233 10.1038/ncb3115

[62] Palchesko RN, Lathrop KL, Funderburgh JL, Feinberg AW. In Vitro Expansion of Corneal Endothelial Cells on Biomimetic Substrates. Sci Rep. Nature Publishing Group; 2015;5: 1743 doi: 10.1038/srep07955 25609008 10.1038/srep07955 PMC4302312

[63] Fenteany G, Janmey PA, Stossel TP. Signaling pathways and cell mechanics involved in wound closure by epithelial cell sheets. Current Biology. 2000;10: 831–838. doi: 10.1016/S0960-9822(00)00579-0 10899000 10.1016/s0960-9822(00)00579-0

[64] Vedula SRK, Hirata H, Nai MH, Brugués A, Toyama Y, Trepat X, et al Epithelial bridges maintain tissue integrity during collective cell migration. Nat Mater. Nature Research; 2014;13: 87–96. doi: 10.1038/nmat3814 24292420 10.1038/nmat3814

[65] Wang L, Sun B, Ziemer KS, Barabino GA, Carrier RL. Chemical and physical modifications to poly(dimethylsiloxane) surfaces affect adhesion of Caco-2 cells. J Biomed Mater Res A. Wiley Subscription Services, Inc., A Wiley Company; 2010;93: 1260–1271. doi: 10.1002/jbm.a.32621 19827104 10.1002/jbm.a.32621

[66] Owen KA, Abshire MY, Tilghman RW, Casanova JE, Bouton AH. FAK regulates intestinal epithelial cell survival and proliferation during mucosal wound healing. Dalmasso G, editor. PLoS ONE. 2011;6: e23123 doi: 10.1371/journal.pone.0023123 21887232 10.1371/journal.pone.0023123 PMC3160839

[67] DiMarco RL, Hunt DR, Dewi RE, Heilshorn SC. Improvement of paracellular transport in the Caco-2 drug screening model using protein-engineered substrates. Biomaterials. 2017;129: 152–162. doi: 10.1016/j.biomaterials.2017.03.023 28342321 10.1016/j.biomaterials.2017.03.023 PMC5572671

[68] Krndija D, Schmid H, Eismann J-L, Lother U, Adler G, Oswald F, et al Substrate stiffness and the receptor-type tyrosine-protein phosphatase alpha regulate spreading of colon cancer cells through cytoskeletal contractility. Oncogene. Nature Publishing Group; 2010;29: 2724–2738. doi: 10.1038/onc.2010.25 20208566 10.1038/onc.2010.25

[69] Ali MY, Saif MTA. Substrate Stiffness Mediated Metastasis Like Phenotype of Colon Cancer Cells is Independent of Cell to Gel Adhesion. Cel Mol Bioeng. 2014;7: 532–543. doi: 10.1007/s12195-014-0345-8

[70] Tang X, Kuhlenschmidt TB, Li Q, Ali S, Lezmi S, Chen H, et al A mechanically-induced colon cancer cell population shows increased metastatic potential. Mol Cancer. BioMed Central; 2014;13: 131 doi: 10.1186/1476-4598-13-131 24884630 10.1186/1476-4598-13-131 PMC4072622

[71] Haage A, Schneider IC. Cellular contractility and extracellular matrix stiffness regulate matrix metalloproteinase activity in pancreatic cancer cells. FASEB J. Federation of American Societies for Experimental Biology; 2014;28: 3589–3599. doi: 10.1096/fj.13-245613 24784579 10.1096/fj.13-245613

