# Supplementary figures and images for: Quantitative assessment of intestinal stiffness and associations with fibrosis in human inflammatory bowel disease
Source: PLoS One. 2018 Jul 11;13(7):e0200377. doi: 10.1371/journal.pone.0200377 (PMC6040714; doi:10.1371/journal.pone.0200377)

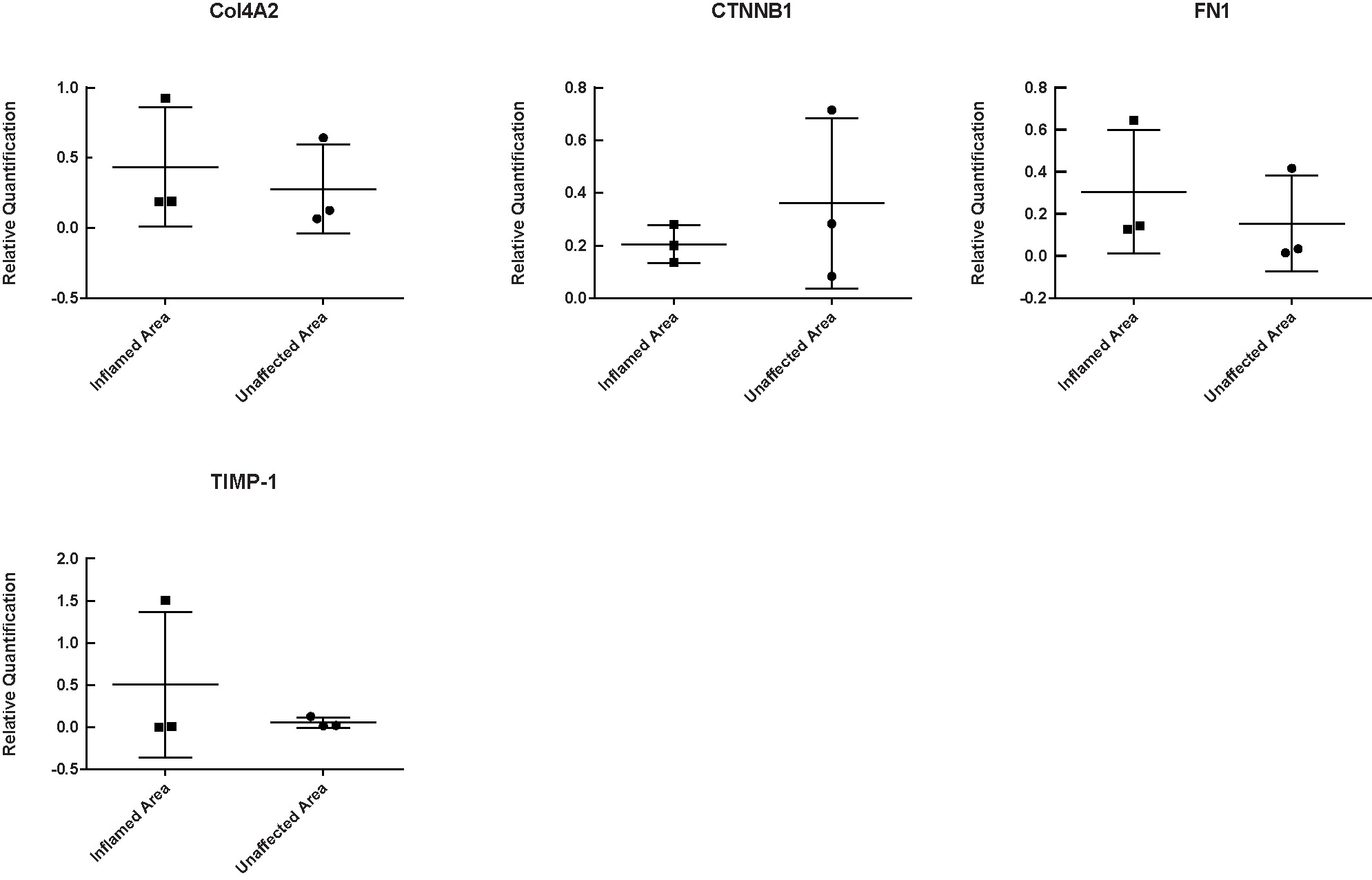

Supplement: S1 Fig — N = 9 per group and is represented as the average of 3 separate experiments. We did not detect significant changes in these genes within our cohort. (TIF) [file pone.0200377.s001.tif]
