# Supplementary material for: Quantitative assessment of intestinal stiffness and associations with fibrosis in human inflammatory bowel disease
Source: PLoS One. 2018 Jul 11;13(7):e0200377. doi: 10.1371/journal.pone.0200377 (PMC6040714; doi:10.1371/journal.pone.0200377)
Supplement: S1 Table — M = Male, F = Female, CD = Crohn’s Disease, UC = Ulcerative Colitis. (DOCX) [file pone.0200377.s002.docx]

| *Patient Number* | *Age* | *Sex* | *Diagnosis* | *Sample Region* | *Pathology by Gross Inspection* |
| --- | --- | --- | --- | --- | --- |
| 1 | 25 | M | CD | Ileum | Inflamed |
| 2 | 42 | M | CD | Ileum | Unaffected |
| 3 | 23 | M | CD | Ileum | Inflamed |
| 4 | 31 | F | CD and adenocarcinoma | Ileum | Unaffected |
| 5 | 46 | F | CD | Ileum | Inflamed |
| 6 | 34 | M | CD | Ileum | Unaffected |
| 7 | 40 | F | CD | Ileum | Inflamed |
| 8 | 33 | M | UC | Ileum | Unaffected |
|  |  |  |  | Colon | Inflamed |
| 9 | 55 | F | UC | Colon | Unaffected |
|  |  |  |  | Colon | Inflamed |
| 10 | 65 | M | CD | Colon | Inflamed |
|  |  |  |  | Ileum | Unaffected |
